# Neural circuits activated by error amplification and haptic guidance training techniques during performance of a timing-based motor task by healthy individuals

**DOI:** 10.1007/s00221-018-5365-5

**Published:** 2018-08-21

**Authors:** Marie-Hélène Milot, Laura Marchal-Crespo, Louis-David Beaulieu, David J. Reinkensmeyer, Steven C. Cramer

**Affiliations:** 1 École de réadaptation, Faculté de médecine et des sciences de la santé, Université de Sherbrooke, Pavillon Gérald-Lasalle, 3001, 12e Avenue Nord, Sherbrooke, QC J1H 5N4 Canada; 2 Sensory-Motor Systems Lab, Institute of Robotics and Intelligent Systems IRIS, ETH Zurich, TAN E3 Tannenstrasse 1, 8092 Zurich, Switzerland; 3 Gerontechnology and Rehabilitation Research Group, ARTORG Center for Biomedical Engineering Research, University of Bern, Murtenstrasse 50, 3008 Bern, Switzerland; 4 Department of Mechanical and Aerospace Engineering, University of California, 4200 Engineering Gateway, Irvine, CA 92697 USA; 5 Department of Biomedical Engineering, University of California, 3120 Natural Sciences II, Irvine, CA 92697 USA; 6 Department of Anatomy and Neurobiology, University of California, 364 Med Surge II, Irvine, CA 92697 USA; 7 Department of Neurology, University of California, 200 S. Manchester AVE, Orange, CA 92868 USA

**Keywords:** Haptic guidance, Error amplification, Brain activation, Motor learning, Timing

## Abstract

To promote motor learning, robotic devices have been used to improve subjects’ performance by guiding desired movements (haptic guidance—HG) or by artificially increasing movement errors to foster a more rapid learning (error amplification—EA). To better understand the neurophysiological basis of motor learning, a few studies have evaluated brain regions activated during EA/HG, but none has compared both approaches. The goal of this study was to investigate using fMRI which brain networks were activated during a single training session of HG/EA in healthy adults learning to play a computerized pinball-like timing task. Subjects had to trigger a robotic device by flexing their wrist at the correct timing to activate a virtual flipper and hit a falling ball towards randomly positioned targets. During training with HG/EA, subjects’ timing errors were decreased/increased, respectively, by the robotic device to delay or accelerate their wrist movement. The results showed that at the beginning of the training period with HG/EA, an error-detection network, including cerebellum and angular gyrus, was activated, consistent with subjects recognizing discrepancies between their intended actions and the actual movement timing. At the end of the training period, an error-detection network was still present for EA, while a memory consolidation/automatization network (caudate head and parahippocampal gyrus) was activated for HG. The results indicate that training movement with various kinds of robotic input relies on different brain networks. Better understanding the neurophysiological underpinnings of brain processes during HG/EA could prove useful for optimizing rehabilitative movement training for people with different patterns of brain damage.

## Introduction

In everyday life, humans often learn to adapt to novel environments or tasks. One paradigm facilitating this process is trial-and-error learning. For this paradigm, studies suggest that execution errors prompt people to adapt by comparing the desired action with the actual action, and finding the best match between sensory information and motor command to accomplish a skillful performance of the task (Halsband and Lange 2006; Nadig et al. 2010). Also, on-line corrections of movement execution errors can occur in the central nervous system with the updating of the internal model of a task to support adaptation and learning (Izawa et al. 2012; Izawa and Shadmehr 2011; Tseng et al. 2007). Studies on learning by trial and error have reported the involvement of several brain regions such as the cerebellum (Hardwick et al. 2013; Heuer and Luttgen 2015), anterior cingulate (Heuer and Luttgen 2015; Taylor et al. 2007), supplementary motor area (Nadig et al. 2010), inferior and superior parietal cortex, dorsolateral frontal cortex (Nadig et al. 2010), and posterior medial frontal cortex (Nadig et al. 2010). A second paradigm facilitating motor adaptation is learning by imitation of an action. Studies suggest that visual and proprioceptive information of a demonstrated action are transformed into a motor output (Buccino et al. 2004; Kessler et al. 2006). This imitation-based learning activates a network of neurons, known as the mirror-neuron system, mainly located in the ventrolateral premotor cortex, posterior and inferior parietal cortex, and superior temporal cortex (Buccino et al. 2004; Caspers et al. 2010; Kessler et al. 2006).

Consistent with these two learning paradigms, i.e., trial-and-error and learning by imitation, robotic devices have been developed to either artificially amplify subjects’ movement errors (error amplification), using force fields (Emken and Reinkensmeyer 2005; Israely and Carmeli 2016; Patton and Mussa-Ivaldi 2004; Patton et al. 2006), visual distortions (Abdollahi et al. 2014) or timing-error amplifications (Bouchard et al. 2015, 2016; Milot et al. 2010), or to guide or demonstrate a correct movement (haptic guidance) to help promote greater learning (Bouchard et al. 2015, 2016; Carel et al. 2000; Ciccarelli et al. 2005; Estevez et al. 2014; Jaeger et al. 2014; Loubinoux et al. 2001; Marchal-Crespo et al. 2013; Milot et al. 2010; Radovanovic et al. 2002). Studies have shown that error amplification (Abdollahi et al. 2014; Bouchard et al. 2015; Emken and Reinkensmeyer 2005; Patton and Mussa-Ivaldi 2004) or haptic guidance (Bouchard et al. 2015, 2016; Marchal-Crespo et al. 2013) training techniques allow improvement in subjects’ performance of the learned task. In healthy individuals, direct comparison of the effectiveness of these two techniques on promoting learning showed that for various upper or lower limb tasks, EA training using either force fields (Marchal-Crespo et al. 2014, 2017b) or visual distortions (van Asseldonk et al. 2009) led to higher learning rate than HG training. However, in a previous study of ours, using a timing-based pinball-like task (identical to the one of the current study), we found that both EA and HG training techniques were beneficial to improving subjects’ timing performance. However, optimized performances were only obtained when individualizing the training technique to best fit subjects’ baseline skill level, that is, EA for the better skilled subjects and HG for the less skilled subjects (Milot et al. 2010).

Little direct comparison has been done on the effect of EA and HG training techniques on brain activation to gain insights into the neurophysiological basis of motor learning. A better understanding of the brain areas activated during both training techniques in healthy subjects could prove useful to help choose which one to apply for people with different types of brain injury, such as a stroke. Indeed, individuals with a stroke can exhibit greater cognitive movement planning time than healthy individuals (Daly et al. 2006) and because of the presence of motor impairments at the affected limb, their timing performance can be twice as long as the one of the unaffected limb (Bi and Wan 2013; Freitas et al. 2011; Miscio et al. 2006), jeopardizing the accomplishment of daily tasks. EA/HG robotic training has been used to help improve timing performance at the chronic phase of a stroke, whereas the side of the brain lesion was shown to influence the response to EA/HG robotic training (Bouchard et al. 2016). A few studies have looked at brain activation related to execution errors during force field (Diedrichsen et al. 2005; Shadmehr and Holcomb 1997), visual rotation (Diedrichsen et al. 2005) and augmented error feedback (Marchal-Crespo et al. 2017a; Nadig et al. 2010) experiments. Studies have reported activation in several brain regions such as posterior medial frontal cortex, cerebellum, superior parietal lobe, and inferior frontal gyrus. During training with EA, it is thought that the posterior medial frontal cortex could play an important role in monitoring errors, while the cerebellar–parietal network could take part in the adjustment of performance in the course of learning (Nadig et al. 2010; Shadmehr and Holcomb 1997). Interestingly, activity in the inferior frontal gyrus has been related to the level of frustration and negative emotion associated with the production of larger than expected errors in the course of training (Nadig et al. 2010). Regarding HG, most studies have evaluated brain activation while subjects remained passive during HG (Carel et al. 2000; Ciccarelli et al. 2005; Estevez et al. 2014; Jaeger et al. 2014; Loubinoux et al. 2001; Radovanovic et al. 2002; Weiller et al. 1996). The sensorimotor cortex was typically activated, possibly due to its involvement in the processing of afferent input (Ciccarelli et al. 2005). In fewer studies, the parietal and temporal cortices, known to be part of the mirror-neuron system (Buccino et al. 2004; Caspers et al. 2010; Kessler et al. 2006), were also activated during passive movements. However, in the previous HG studies, subjects were requested to remain passive while the robot performed the movement, and therefore, it is unclear whether brain areas associated with learning by imitation should also be activated when the subjects are actively involved in the movement generation.

Following our behavioral study in healthy young subjects (Milot et al. 2010), we conducted an imaging experiment to gain insight into the learning circuits specific to training with EA and HG during learning of a timing task in healthy young subjects, knowing that timing is an essential prerequisite to movement execution (Georgopoulos 2002) and that it can be impaired after a stroke (Bi and Wan 2013). The EA and HG strategies were designed to augment or reduce the timing errors, respectively. HG did not eliminate the errors completely, and therefore, subjects had to actively perform the task, independently of the robotic strategy used during training. Based on the literature, it seems that EA and HG promote learning based on different putative brain networks. Thus, for a timing-based task, we hypothesized that training with EA would activate different brain areas related to learning as compared to training with HG. We hypothesize to find activity in somatosensory/motor related areas (S1/M1) and supplementary and pre-supplementary motor areas (SMA/pSMA) when training with HG and EA. Specifically, we hypothesized that training with EA would translate into activation of brain regions associated with error-based learning, such as the cerebellum and anterior cingulate. We expected weaker sensory/parietal activity when training with HG. Hypothetically, the mirror-neuron system (such as the ventrolateral premotor cortex and parietal cortex) might be implicated during training when the robot haptically guides subjects to actively perform the task with smaller errors.

## Materials and methods

### Subjects

Eighteen healthy subjects (10 female; 8 male) with a mean age of 22.3 ± 3.4 years were recruited from the student population of the University of California, Irvine (UCI). The number of subjects to be included in this study was determined based on the significant differences found between the HG and EA strategies in a behavioral study performed with 20 young healthy subjects (Milot et al. 2010) using a timing-based pinball-like task (identical to the one of the current study). To be included in the study, subjects had to be right-handed (Edinburgh handedness questionnaire mean score: 85 ± 15%), have no active neurological or orthopedic problem affecting the right upper extremity and be able to undergo an MRI scan. Informed consent was obtained from each subject before the evaluation session, and the UCI Institutional Review Board approved the study.

### Motor learning task

**Fig. 1:**
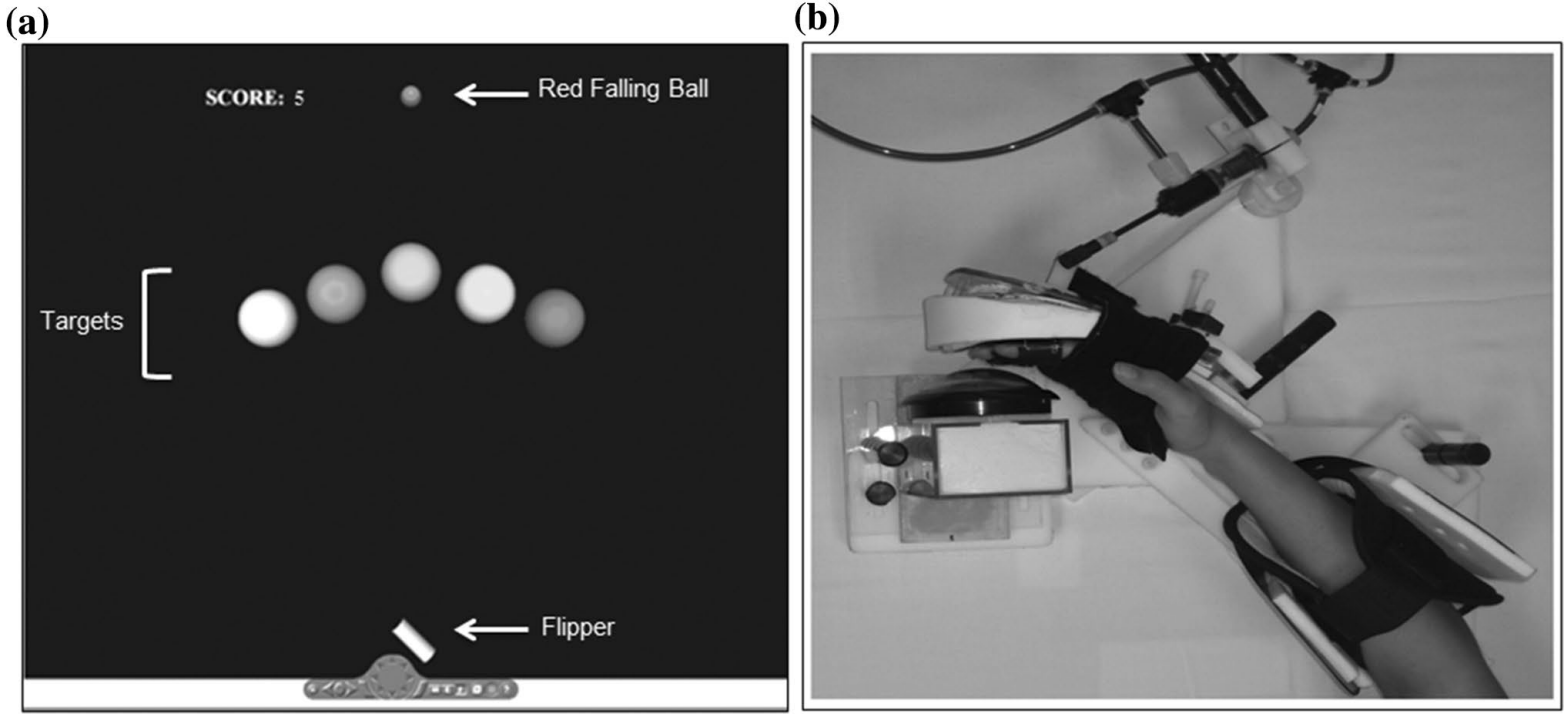
**a** The video display of the pinball game showing the position of the five targets, the score, the falling ball and the flipper. **b** The hand/robot interface component of TAPPER was actuated by air pressure and rotated the subject’s wrist so that the fingers would depress the button to produce a flipper rotation

Subjects had to learn a pinball-like game that was identical to the game described by our group previously [for more details see (Milot et al. 2010)]. In sum, while viewing a computer screen, subjects placed their right hand in Timing Assistive Plastic Pinball Exercise Robot (TAPPER) (Fig. 1). They then had to move their hand with the proper timing to hit a falling ball towards a presented virtual target. TAPPER is a MRI-compatible pneumatic one-degree-of-freedom plastic robot. It is composed of a forearm brace mounted on a frame, a freely rotating hand brace connected to a pneumatic cylinder, and a button that is depressed by the subject’s fingers when the hand/robot unit rotates in wrist flexion (see Fig. 1b). The pinball-like game presented on the computer screen consisted of a falling ball, a flipper and five targets, presented one at a time randomly and positioned at different location across the computer screen (Fig. 1a).

The video display of the game was projected onto a transparent screen inside the MRI tunnel and viewed by the subjects through a mirror mounted on the MRI head coil. Subjects were instructed to time their hand movements such that as many targets as possible would be hit, by triggering wrist flexion, to overcome the 1 psi resistance of the locked pneumatic cylinder. The subjects’ wrist flexion translated into a small wrist excursion of about 4 mm. This made TAPPER’s pneumatic cylinder move, which then caused TAPPER to produce 5 degrees of wrist flexion, moving the subject’s hand into contact with the button. When this button contact did occur, it produced flipper rotation on the computer screen, and the flipper would then hit the falling ball depending on timing. Each target was related to a specific desired time of wrist flexion initiation for the ball to hit the target. A new target appeared every 2 s. Trials were considered successful when the flipper hit the ball in such a manner as to result in the ball hitting the target (within ± 6.9°), which corresponded to a button press timing accuracy of 4 ms. For each successful trial, a 1-point reward was given. Also, on each trial, a visual feedback was presented to the subjects to notify them about their timing error and instruct them on what action to take on the subsequent trial (“Wow! Just on time!”; “Too early. Hit later!”; “Too late. Hit sooner!”).

### Algorithms used to provide error amplification/haptic guidance and adjust the game difficulty

A detailed description of the algorithms used to provide error amplification and haptic guidance was presented in our previous work (Milot et al. 2010). In sum, for error amplification, we wanted to increase subjects’ timing error by delaying or speeding up the start of the robot movement if the subject initiated a wrist flexion too late or too early, respectively. For haptic guidance, we desired to minimize subjects’ timing error by speeding up or delaying the start of the robot movement if the subject initiated a wrist flexion too late or too early, respectively. We wanted the resulting timing error (*E*_b_) to be proportional to the subjects’ timing error (*E*_p_) with a proportionality constant *k*, called the error amplification gain (Eq. 1):

1 $${E_{\text{b}}}=k{E_{\text{p}}}.$$

To achieve this, we used Eq. 2 to set the delay (*D*_c_) between the moment the subject initiated a wrist flexion movement and when TAPPER began to move, proportionally decreasing or increasing subjects’ timing error:

2 $${D_{\text{c}}}~=~D{c_{\text{d}}}+{E_{\text{p}}}(k - {\text{1}}).$$

Note that *D*c_d_ was a constant 0.5-s delay between when the subjects triggered a wrist flexion and when the pneumatic cylinder moved, making subject’s fingers to contact the flipper-activating button. Subjects had to take that delay into consideration while playing the pinball-like game. A value of *k* = 1 resulted in no error amplification or haptic guidance; a value of *k* > 1 increased timing errors (error amplification); a value of *k* < 1 attenuated errors (haptic guidance), and a value of *k* = 0 would theoretically result in the subject always hitting the target independent of their timing error (as long as *E*_p_ < *Dc*_d_).

Before providing error amplification or haptic guidance, we wanted to adjust the level of difficulty of the task to each subject skill level to achieve a similar baseline performance across subjects, and control for the effect of skill level (Milot et al. 2010) and task difficulty (Guadagnoli and Lee 2004) on learning. The adjustment of the level of difficulty was based on the subjects’ timing error and the desired rate of success to be reached (set at 30%) and determined as follows:

3 $$k(i+1)={g_1} \cdot k(i) - {g_2} \cdot \left[{w_1} \cdot (R{s_{\text{d}}} - R{s_{\text{p}}})+{w_2} \cdot \left| {T{b_{\text{p}}} - T{b_{\text{d}}}} \right|\right],$$

where *g*_1_ (1.02) and *g*_2_ (0.15) represented learning gains, and were weighted gains (*w*_1_ = 0.25 and *w*_2_ = 0.9). *Rs*_p_ and *Rs*_d_ were the subject and desired rate of success, respectively, whereas *Tb*_p_ represented the time the subject’s fingers actually press the button and *Tb*_d_ was the desired time the subject fingers should have press the button. This adjustment of the game difficulty was made after each trial during a baseline condition, B2 (see section below). In a previous behavioral study with 20 healthy participants who underwent a similar experimental protocol, the game difficulty adjustment using Eq. 3 during B2 systematically reduced the task difficulty until reaching a success level of 23 ± 14% (Milot et al. 2010).

### fMRI protocol

Using the SENSE coil, the subject’s head was stabilized with padding and a strap to minimize head motion during scanning. A foam pad was placed on the subject’s abdomen and a wedge was stabilized on it with Velcro. The wedge allowed proper angle, positioning and stabilization of TAPPER on the subject’s abdomen. The subject’s forearm and hand were positioned inside the brace component of TAPPER, and then the MRI response box was stabilized with plastic screws behind the robot button. Careful attention was provided to make sure that the pieces were aligned such that a push on the TAPPER button consistently produced a push on one of the 4 buttons on the MRI response box. The signal was read by the fORP Electronic Interface unit (Current Designs, Inc., Philadelphia, PA, USA) using a TTL connection at a frequency of 1000 Hz. Pressure tanks provided air to the robot with tubing inserted through the designated slot in the MRI room wall.

MRI data were collected with a 3-T Philips scanner. First, high-resolution T1-weighted anatomical images were acquired in sagittal orientation (TR 8.1 ms; TE 3.7 ms; flip angle 8°; number of slices 160; resolution 1 × 1 × 1 mm^3^). Second, fMRI data were obtained by use of gradient echo planar T2*-weighted imaging collected in axial orientation (TR 2000 ms; TE 30 ms; flip angle 70°; number of slices 29, gap between slices 1 mm; slice thickness 4 mm; in-plane resolution 3 × 3 mm^2^). The MRI scanner was activated during the baseline condition as well as during each training condition.

### Study design

**Fig. 2:**
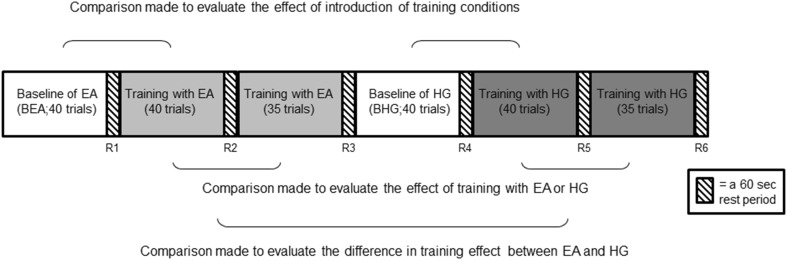
Study design showing an example of a subject training first with error amplification (EA), followed by haptic guidance (HG). Note that half the subjects were randomized to have HG training precede EA training. *R* rest

Brain activation was evaluated using a within-subject crossover design. The TAPPER pinball game was divided into three parts (baseline condition, training condition 1, and training condition 2; see Fig. 2) and synchronized with the MRI scanning using Matlab^®^.

The baseline condition started with 39 trials played with an error amplification gain (*k* value) of 1, i.e., no error condition was provided (Baseline 1: B1). These trials served as a familiarization phase for subjects to become accustomed to the device, the timing task, and the 0.5-s delay. Afterwards, another 39 trials were used to gradually adjust the *k* value using Eq. 3 (Baseline 2: B2). The greater the final *k* value at the end of the 39 trials, the higher subject’s skill level was. Note that B1 and B2 (which are not shown in Fig. 2) were not further analyzed, e.g., for brain activation. The last part of the baseline condition was composed of 40 trials (shown in Fig. 2) and was played at the adjusted difficulty level (*k* value) that was established during the adjusting baseline period (B2). These 40 trials were used as the subject’s baseline timing performance and brain activation status and were followed by a 1-min rest (R1). During rest, subjects were instructed to remain motionless while watching an ‘X’, instead of a ball, fall on the screen. Subsequent rest periods of the study design were identical to R1. The total duration scan of the baseline condition was around 12 min.

For the two training conditions (EA and HG), the order of presentation to subjects was randomized and not revealed to subjects. Using Eq. 2, the final adjusted *k* value for each subject was increased or decreased by 90% for error amplification and haptic guidance, respectively. This change in the individual *k* value remained constant throughout EA/HG training and has been shown to be sufficient to create a significant change in subjects’ timing error between training conditions (Milot et al. 2010). Each training condition contained 75 trials with a 1-min rest (R2 and R5) after the first 40 trials and at the end of the training condition (R3 and R6). The total scan duration of each training condition was around 12 min.

### Statistical analysis

For the behavioral analysis, the main dependent measure was absolute timing error. This was calculated as the absolute difference between when subject pressed the button and the optimal time at which the subject should have done so, based on the robotic software and hardware. The secondary outcome measure was relative timing error, with a negative value indicating a delayed wrist movement initiation. Normality of data was assessed with the Kolmogorov–Smirnov test. Both the absolute and relative timing error datasets were not normal and for the latter, it could not be transformed. Thus, Wilcoxon signed-rank tests, with a Bonferroni correction for running multiple tests, were used to evaluate: (a) the presence of a learning plateau at the end of the baseline condition, (b) the effect of introducing EA and HG training conditions, respectively, on timing error, and (c) the change in timing error from the beginning to the end of training with EA and HG, respectively. The threshold for significance was set at an adjusted *p* value of 0.025. All statistical analyses were performed using SPSS® software Windows (version 13, Chicago, IL, USA).

For the fMRI analysis, images were realigned to the first image, coregistered, and normalized to the MNI reference brain. Data were smoothed using a Gaussian kernel of 8-mm full width at half maximum. All data were visually inspected to confirm absence of head motion artifact, and subjects with head movement exceeding 3 mm of translation on the *x, y, z* axis or 3° of rotation were excluded; one subject had to be excluded on this basis. All fMRI analyses were performed with SPM5 (http://www.fil.ion.ucl.ac.uk/spm/). For both EA and HG training, within-subject contrast images were created for three conditions: contrast [1], the last 10 trials of baseline vs. rest, done twice for each subject (once for the baseline for EA [BEA] and once for the baseline for HG [BHG]), thus BEA > R1 and BHG > R4 (or BHG > R1 and BEA > R4, depending on order of training to which subject was randomized); contrast [2], the first 10 trials of training vs. rest, thus EA > R2 or HG > R5; and contrast [3a], the first 10 trials > the last 10 trials of each training, for EA and for HG, respectively, and contrast [3b], the last 10 trials > the first 10 trials of EA and HG, respectively. These contrast images were entered into between-subject analysis with significance defined at an uncorrected threshold of *p* ≤ 0.001. To evaluate the effect that introduction of training with EA or HG had on brain activation, a paired t-test was used to estimate contrast [2]–contrast [1], for both EA and HG, respectively. To determine changes in brain activation across the period of training with EA or HG, t-tests were performed on contrast [3a] and [3b], separately for EA and for HG, respectively. Finally, to directly test for differences in brain activation between the two training conditions, contrast [3a] for EA was compared with contrast [3a] for HG, as well as the reverse contrast, using paired t-tests.

## Results

### Behavioral data

At baseline, across all subjects, when the first 10 and last the 10 of the final 40 baseline trials were compared, no significant change was observed in absolute (*p* = 0.57) and relative (*p* = 0.74) timing errors. Therefore, a learning plateau was reached before the introduction of the training conditions. Regarding introduction of EA, when comparing the last 10 trials of BEA to the first 10 trials of EA, absolute timing error significantly increased (16 ± 21 vs. 26 ± 22 ms; *p* = 0.002), with subjects tending to initiate wrist movement later (− 6 ± 17 vs. − 13 ± 21 ms; *p* = 0.09). Conversely, regarding introduction of HG, when comparing the last 10 trials of BHG to the first 10 trials of HG, timing error significantly decreased (13 ± 9 vs. 9 ± 16 ms; *p* = 0.014), but subjects relative timing error did not change because in both phases, it was already close to zero (1 ± 10 vs. 0.4 ± 12 ms; *p* = 0.42). Thus, introduction to the EA and HG conditions produced the expected effect on timing performance.

**Fig. 3:**
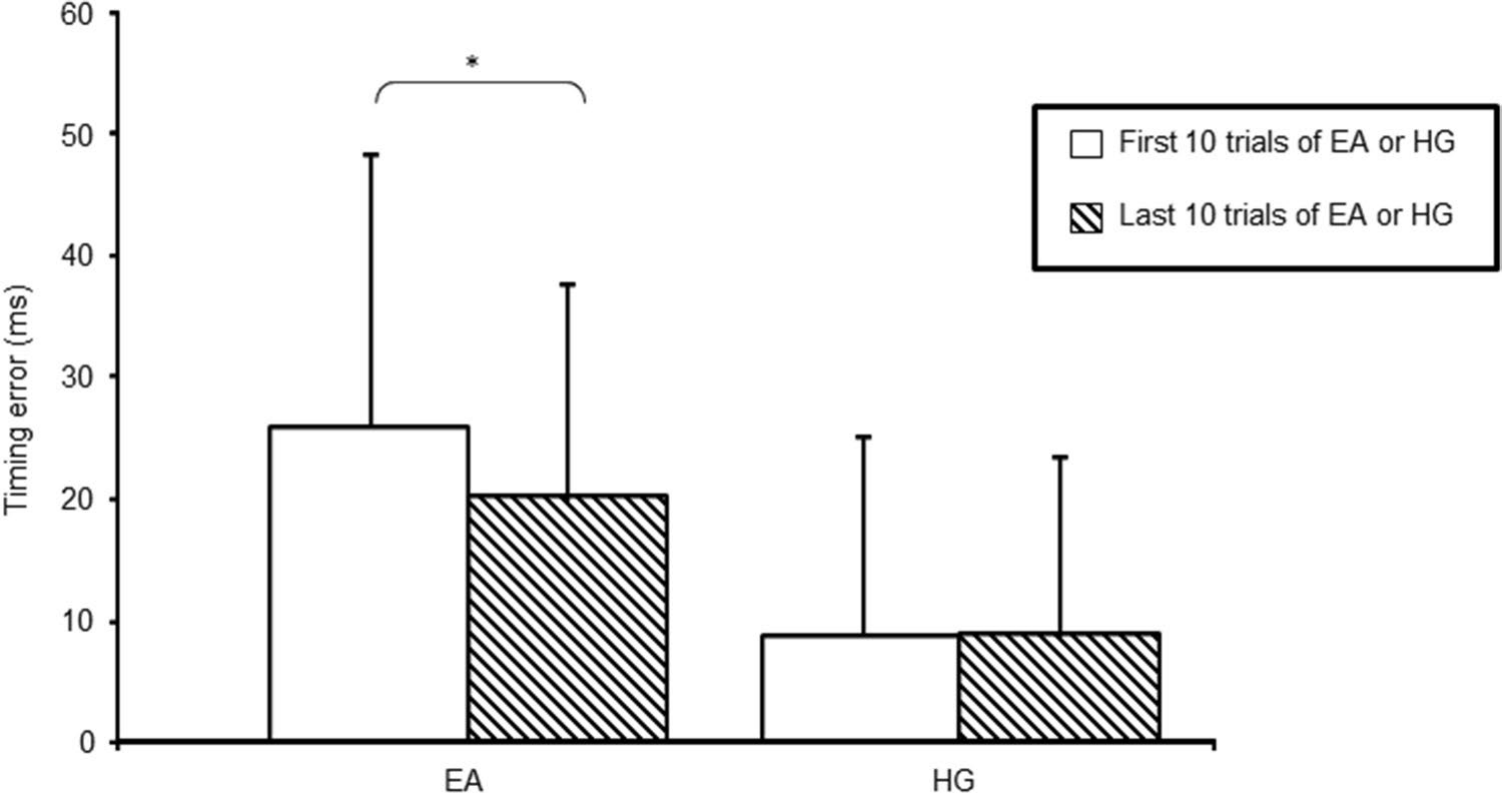
Comparison of absolute timing error between the first and last 10 trials during training with error amplification (EA) and haptic guidance (HG). Error bars show ± 1 SD. *p < 0.05

Short-term learning occurred across the period of error amplification training, as improvement in absolute timing error was noted when comparing the first with the last 10 trials of this condition (27 ± 22 vs. 20 ± 17 ms; *p* = 0.02). When looking at the relative timing error, no change was noted in subjects’ performance meaning that they still initiated wrist movement too late (− 13 ± 21 vs. − 8 ± 17 ms; *p* = 0.14). However, no learning was noted across the period of haptic guidance training (9 ± 16 vs. 8 ± 15 ms; *p* = 0.28), with subjects relative timing error staying close to zero (0.4 ± 12 vs. 2 ± 10 ms; *p* = 0.36). As mentioned in our previous work (Milot et al. 2010), application of HG creates a floor effect such that timing error is too low to detect a training-related benefit (see Fig. 3).

### Imaging data

#### Effect of introduction of training with EA or HG on brain activation

**Fig. 4:**
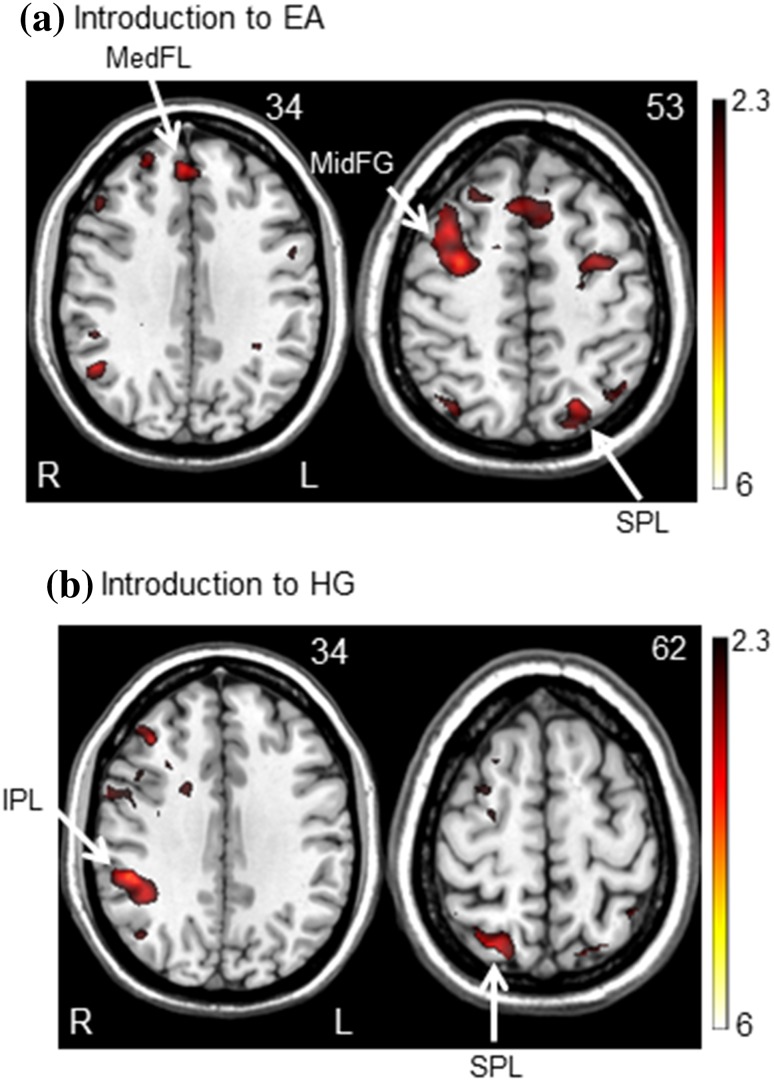
Group maps of brain activation during introduction to **a** error amplification (EA) and **b** haptic guidance (HG) training conditions. The Montreal Neurological Institute *z* values (right corner of each image) represent the displayed axial slice level along the dorsal/ventral axis. *MidFG* middle frontal gyrus, *MedFL* medial frontal lobe, *IPL* inferior parietal lobe, *SPL* superior parietal lobe

Table 1 lists the areas activated when subjects were introduced to EA and HG training conditions, as compared to their respective baselines (i.e., contrast [2]–contrast [1]). Introduction to EA significantly activated the left superior parietal lobe, right middle frontal gyrus, and right medial frontal lobe; while introduction to HG significantly activated the right superior parietal lobe and inferior parietal lobule (see Fig. 4).

**Table 1:** Regions of significant activation when contrasting EA or HG relative to their respective baselines

Region	Side of brain	Peak *Z* score	*x*	*y*	*z*	Cluster volume^a^
Introduction to EA						
Superior parietal lobe	l	4.69	− 22	− 68	62	48
Middle frontal gyrus	r	4.26	32	0	56	152
Middle frontal gyrus	r	4.06	36	14	50	134
Medial frontal lobe	r	3.67	4	46	34	35
Introduction to HG						
Superior parietal lobe	r	4.08	26	− 66	62	53
Inferior parietal lobule	r	4.04	50	− 38	34	200

*r* right, *l* left

^a^The clusters are significant at *p* ≤ 0.05, and the cluster volume is reported in 2 mm^3^ voxels

#### Changes in brain activation across the period of training

Greater activation was found at the beginning of EA training, as compared to the end of EA training (contrast [3a]), within bilateral cerebellum and angular gyrus as well as in the left superior frontal gyrus. On the other hand, greater activation was found at the end of EA training, as compared to the beginning of EA training (contrast [3b]), within right anterior cingulate and left superior temporal gyrus.

Greater activation was found at the beginning of HG training, as compared to the end of HG training (contrast [3a]) within bilateral cerebellum, right angular gyrus and right middle frontal gyrus. However, greater activation was noted at the end of HG training, as compared to the beginning of HG training (contrast [3b]), within left caudate and right parahippocampal gyrus.

#### Differences in brain activation between training with EA and HG

**Fig. 5:**
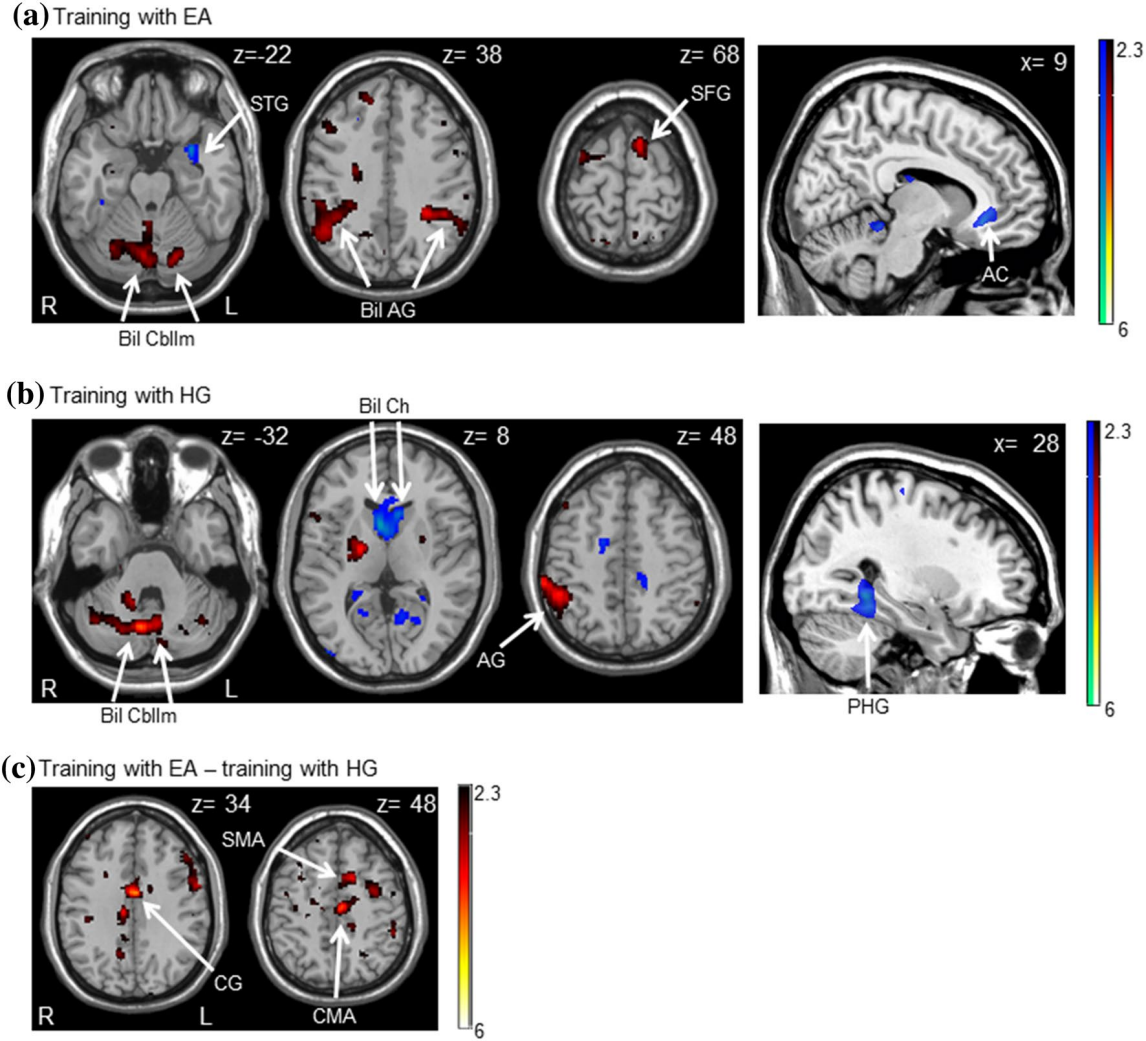
Group maps of greater brain activation at the beginning–end of training [red activation] and end–beginning or training [blue activation] with **a** error amplification (EA) and **b** haptic guidance (HG), and **c** group maps of brain activation of the difference between training with EA and HG. The Montreal Neurological Institute *z* and *x* values (right corner of each image) represent the displayed axial slice level along the dorsal/ventral and/or right/left axis, respectively. *AC* anterior cingulate, *AG* angular gyrus, *Bil AG* bilateral angular gyrus, *Bil Cbllm* bilateral cerebellum, *Bil Ch* bilateral caudate head, *CMA* cingulate motor area, *CG* cingulate gyrus, *PHG* parahippocampal gyrus, *SMA* supplementary motor area, *STG* superior temporal gyrus, *SFG* superior frontal gyrus

EA and HG were directly contrasted. Training with EA (beginning of EA training compared to the end of EA training), as compared to training with HG (beginning of HG training compared to the end of HG training) caused significant activation within left cingulate motor area, cingulate gyrus, and supplementary motor area. No significant activation was found when computing the reverse contrast (see Table 2; Fig. 5).

**Table 2:** Region of significant activation across the training conditions (EA: a, b; HG: c, d) and differences in activation between both EA and HG (e, f)

Region	Side of brain	Peak *Z* score	*x*	*y*	*z*	Cluster volume^a^
(a) First 10 trials EA > last 10 trials EA						
Cerebellum	r/l	6.73	4	− 72	− 30	372
Angular gyrus	r/l	4.04	50	− 34	44	312
Superior frontal gyrus	l	3.29	− 10	8	64	60
(b) Last 10 trials EA > first 10 trials EA						
Anterior cingulate	r	4.35	8	30	− 10	85
Superior temporal gyrus	l	4.09	− 32	6	− 28	66
(c) First 10 trials HG > last 10 trials HG						
Angular gyrus	r	3.57	50	− 48	52	267
Cerebellum	r/l	4.29	6	− 68	− 34	157
Middle frontal gyrus	r	6.49	48	40	− 8	73
(d) Last 10 trials HG > first 10 trials HG						
Caudate head	r/l	4.57	− 10	16	10	398
Parahippocampal gyrus	r	4.19	28	− 46	− 6	113
(e) [First 10 trials EA > last 10 trials EA] > [first 10 trials HG > last 10 trials HG]						
Cingulate motor area	l	4.09	− 4	− 22	50	103
Cingulate gyrus	l	4.62	− 2	− 4	32	72
Supplementary motor area	l	3.81	− 14	10	48	56
(f) [First 10 trials HG > last 10 trials HG] > [first 10 trials EA > last 10 trials EA]	No suprathreshold clusters					

*r* right, *l* left

^a^The clusters are significant at *p* ≤ 0.05, and the cluster volume is reported in 2 mm^3^ voxels

## Discussion

This study directly compared the short-term impact of EA and HG training conditions for learning a timing-based task on brain activation in healthy young subjects. Based on the literature, we hypothesized that an error-detection network would be more strongly activated by training with EA, whereas training with HG would activate brain regions related to learning by imitation. Overall, the results support our hypotheses for EA, but not for the HG training condition.

### Introduction to EA and HG training conditions

When subjects were introduced to the EA training condition, the current results show the activation of a fronto-parietal motor attention network (Rushworth et al. 2001; Scolari et al. 2015) involved in the preparation of a motor response (Rushworth et al. 2001) as well as in the adjustment of the motor response (Japee et al. 2015; Nadig et al. 2010). Both the middle frontal gyrus and medial frontal lobe were activated. Regarding the middle frontal gyrus, with the unexpected increase in subjects’ timing errors from their baseline to their EA condition, subjects had to readjust their performance to meet the requirement of the EA training condition and they did so by activating the middle frontal gyrus, involved in the reorientation of a person’s attention to unexpected stimuli (Japee et al. 2015). For the medial frontal lobe activation, a previous study found a significant relation between the magnitude of errors and the degree of activation in this brain region (Nadig et al. 2010). With the increase in the subject’s timing errors during EA, the activation of the medial frontal lobe may relate to signaling the motor system the need to change the subjects’ performance in order to succeed at the task (Nadig et al. 2010); as noted by the improvement in the subjects’ timing performance across the EA training condition. Since introduction of HG training did not cause an increase in the subjects’ timing errors, and consequently the need to readjust the motor response as much as in the EA training condition, this could explain the lack of involvement of these frontal brain regions.

However, for both training conditions, parietal regions known to be involved in the processing of timing (Battelli et al. 2007) and movement planning (Desmurget et al. 2009) were activated, but on the left hemisphere for EA and the right hemisphere for HG, the latter presenting a large cluster of activation. For the EA condition, in their review on the role of the parietal cortex, Daprati et al. (2010) have reported the lack of ability to execute complex movement in subjects presenting a left parietal brain lesion. This could be explained by the crucial role played by this brain area in encoding spatial and temporal movement information (Land 2014; Rugg and King 2017; Sirigu et al. 1999) in order to help the formation of an internal model of the task or movement to perform, important for motor learning. Indeed, in a previous paper of ours, we showed that a crucial aspect of learning a timing-based task was the capability to form an internal model of the delay associated with the triggered movement (Milot et al. 2010). Thus, during EA, where timing errors are increased, trying to form an internal model of the correct timing could be even more essential for learning due to the increased difficulty of the task. During HG, since the subjects’ timing errors were small, the formation of an internal model of the timing task could have been less essential for learning and only the right parietal cortex was activated, possibly due to its role played in visuospatial processing (Battelli et al. 2007; Daprati et al. 2010; Rushworth et al. 2001). Notably, when looking at Table 1, an important cluster of activation was noted in the inferior parietal lobe for the HG condition. A study by Farrer et al. (2008) have shown activation of this brain region when a discrepancy was introduced between their participants’ action and the feedback provided during a peg removal task. This is also the case in the current study since the introduction to the HG condition translated into a sudden drop in the subjects’ timing errors as compared to their baseline performance, leading to a discrepancy between the subjects’ performance during the baseline condition compared to their performance when introduced to HG.

In brief, introducing subjects to EA activates a fronto-parietal network to address the sudden increase in timing errors. For HG, since timing errors become small, only a parietal activation is noted, involved in the process of timing and movement planning.

### Changes in brain activation across the period of EA and HG robotic training

When comparing the brain regions activated at the beginning as compared to the end of training, two large clusters of activation were found bilaterally in the cerebellum and angular gyrus for EA as well as bilaterally in the cerebellum and in the right angular gyrus for HG robotic training condition. Regarding the cerebellum, the results showed a bilateral activation for both training conditions supporting the widely accepted role of the cerebellum in the learning of a new motor skill, that is, to predict the sensory consequences of a motor task and detect errors between these predictions and the actual sensory feedback. The cerebellum is in fact recognized as a key structure involved in the formation and update of internal models of movements [for reviews, see (Hardwick et al. 2013; Heuer and Luttgen 2015; Izawa et al. 2012; Izawa and Shadmehr 2011; Makino et al. 2016; Shadmehr and Holcomb 1997; Tseng et al. 2007)]. A recent meta-analysis of 70 experiments (Hardwick et al. 2013) reported a bilateral activation of the cerebellum during the learning of various hand tasks (both unimanual and bimanual tasks were pooled in the analyses) and underscored that the cerebellum might be more active during the initial phases of motor learning, as in the current study.

Similarly to the cerebellum, the right angular gyrus was activated in both training conditions. This brain structure is involved, among other functions, in the conscious awareness of a person’s own action, by monitoring the discrepancies between the planned and resulting movement (Chambon et al. 2014; Farrer et al. 2008) and most likely implies explicit learning mechanisms. For example, Farrer et al. (2003) reported a significant correlation between the level of activation of the right angular gyrus and the magnitude of errors between the intended movement and the actual sensory consequences. Indeed, the subjects were first trained to control a virtual hand with a joystick, hence enabling them to learn the link between the planned movements and their consequences. Then, distortions were introduced experimentally to the virtual hand, resulting in a significant activation of the subjects’ right angular gyrus, which was strongest under greater distortions (Farrer et al. 2003). The authors argued that an activation of the right angular gyrus is only detectable when the sensory feedback is experimentally manipulated, as in the current study. The introduction to both EA and HG thus yielded a significant discrepancy in the subjects’ magnitude of timing errors as compared to their baseline condition, and the subject’s awareness of such errors was most likely processed by the right angular gyrus.

The left angular gyrus was also active during the first vs. the last trials of robotic training, but only for the EA condition. The larger timing errors during EA as compared to HG resulted in a greater discrepancy between the subjects planned action and their actual timing performance, which might explain why the angular gyrus was bilaterally active during EA but not during HG. On the other hand, the left angular gyrus activation is also related to speech processing mechanisms linked to the comprehension of visual words/sentences (Price 2010; Roux et al. 2015). It can be hypothesized that the difference between EA and HG training in the activation of the left angular gyrus originates from the visual feedback presented to the subjects about their performance. Under HG training, even if the subjects’ actual movement timing was too early or too late, the ball hit the target at the right time during most of the training. Hence the visual feedback “Wow! Just on time!” was almost always presented to the subjects after each trial. Conversely, during EA, the subjects timing errors were artificially increased and thus they were rarely on time to hit the targets and the visual feedback “Too early. Hit later!” or “Too late. Hit sooner!” were frequently displayed on the computer screen. Therefore, left angular gyrus activation could have contributed to the interpretation of this visual feedback as it was used to update the motor program and promote short-term motor learning, which occurred to a greater extent during EA rather than HG training.

When contrasting the end vs. the beginning of each training condition, small clusters of activation were noted for EA in the right anterior cingulate and left superior temporal gyrus (Table 2). The anterior cingulate cortex is acknowledged to be part of a broader error-detecting system (Heuer and Luttgen 2015; Taylor et al. 2007), and its rostral subsection processes the emotional response of an error signal [reviewed in Taylor et al. (2007)]. During training with EA, the current subjects could have experienced frustration or disappointment because of the increased task difficulty that translated into large timing errors. The left superior temporal gyrus is a key structure involved in reading comprehension (DeWitt and Rauschecker 2013; Friederici et al. 2003; Price 2010; Roux et al. 2015). Importantly, previous work showed a significant activation of the left superior temporal gyrus when reading action-related sentences (Kana et al. 2015), such as the visual cue provided during the timing task, hence further supporting that subjects might have relied on this feedback to try adjusting their motor program in the course of EA training.

At the end of HG practice, two significant clusters of activation were observed in the right parahippocampal gyrus and bilateral head of the caudate nucleus, which was particularly large for the latter. The activation of these brains structures suggests that a memory consolidation of the motor program related to the timing task was ongoing at the end of the HG practice. Movement execution could have relied on memory consolidation, even after only 75 trials of training with HG, possibly because during HG, timing errors were small and movement execution was almost perfect, hence there was no need to activate a complex error-detection/correction network such as during EA (Kim et al. 2011; Luck et al. 2010; Owen et al. 1996). In addition, the caudate nucleus, a core component of the striatum (Helie et al. 2015), is known to be involved in several processes related to motor learning and reward, including motor program automatization during the latter stages of learning (Park et al. 2010), automatic behavior (body of caudate) and cognitive control of action (head of caudate) (Ashby and Crossley 2012; Kim and Hikosaka 2015; Morris et al. 2016). For instance, the basal ganglia can reinforce the cortico-cortical networks that produced the correct behavior through Hebbian learning, ultimately leading to development of automatic behaviors (Helie et al. 2015). This process is also related to reward-based learning, because the basal ganglia [particularly the caudate head—see meta-analysis by Arsalidou et al. (2013)] is activated in response to a reward (Nakamura et al. 2012; Schultz 2016). Heuer and Luttgen (2015) recently postulated that haptic guidance training can promote reward-based learning because of the high success rate (= reward) related to it. More precisely, dopamine neurons in the striatum are activated during training in response to a reward or to reward-predicting stimuli and can strengthen synapses to promote actions that lead to the reward (Heuer and Luttgen 2015; Schultz 2016). This could explain the large cluster of activation observed in the bilateral caudate head while learning the timing task with HG.

In short, in the beginning of training with EA and HG, bilateral activation of the cerebellum was noted, possibly due to its key role in the learning of a new motor task. The angular gyri were also activated in both training conditions, but to a larger extent during EA, possibly due to the subjects’ awareness of their timing errors and their reliance on the feedback provided while training to adapt to the task. By the end of training with EA and HG, an error-detection network, involving the cingulate cortex, was activated with EA while HG involved mainly a reward-based learning probably because the subject’s success rate at hitting targets was quite high.

### Differences in brain activation between training with EA and HG

When contrasting the changes of brain activation between EA and HG throughout training (i.e., first 10 vs. last 10 trials), significant clusters were found for the left cingulate cortex and left supplementary motor area (Table 2) indicating that these structures were more strongly activated at the beginning of EA than HG training conditions. The cingulate cortex is part of the limbic system and as mentioned above, it contributes to an error-detecting network processing the emotional/motivational response to an error and signaling the need to adapt the behavior when the action failed to produce the desired effect, such as during EA (Heuer and Luttgen 2015; O’Connell et al. 2007; Taylor et al. 2007). On the other hand, the supplementary motor area is a key structure involved in motor planning and was likely more strongly activated under EA training possibly for updating the motor program from trial to trial in response to the large timing errors (Halsband and Lange 2006). Interestingly, the left supplementary motor area has been found to be particularly activated during the training of motor skills requiring a precise timing or rhythm [see review by Halsband and Lange (2006)], similarly to the present work. In addition, we first hypothesized that EA would translate into a greater activation of the cerebellum than HG due to the presence of large timing errors during EA training. The absence of activation of this brain area during EA as compared to HG could be related to the fact that, at the beginning of both training conditions, the cerebellum is highly active due to its role played mainly in the initial phase of learning, as mentioned previously. Thus, when contrasting brain areas activated during EA to those during HG, no difference in activation in the cerebellum was noted.

Conversely, no cluster of activation was found when contrasting HG vs. EA, likely because HG training was less challenging than EA and thus involved activation of fewer brain regions. Lower brain activation during HG has been acknowledged in other studies comparing active vs. HG during passive movements (Ciccarelli et al. 2005; Jaeger et al. 2014; Loubinoux et al. 2001). Hence, it can be hypothesized that this less brain activation, as opposed to the activation of a more complex error-detection brain network with EA, could explain in part the lack of a generalization of motor learning to other tasks or untrained targets, often observed following HG (Heuer and Luttgen 2015; Milot et al. 2010).

In sum, differences of activation between EA and HG rely mainly in the activation of brain areas related to the detection of errors and motor planning for EA.

### Why HG did not activate the mirror-neuron system?

We expected that HG training would more strongly activate brain areas related to the mirror-neuron system, such as ventrolateral premotor cortex and parietal cortex (Buccino et al. 2004; Caspers et al. 2010; Kessler et al. 2006). However, our results are not in line with this hypothesis. One reason could be that previous studies evaluating the mirror-neuron system focused on tasks involving mainly action observation (Buccino et al. 2004; Caspers et al. 2010; Kessler et al. 2006). In our study, however, the vision of the hand (and the robot) was occluded by the mirror mounted on the MRI head coil to show the game video display. Therefore, subjects only saw the flipper moving after pressing the button once the wrist flexion movement was completed.

We hypothesized, however, that proprioceptive information provided by the robot during HG would lead to similar activation of the mirror-neuron system as action observation. A possible rationale for the lack of enrollment of the mirror-neuron system might originate from the compliant nature of the HG employed in our experiment. Contrary to previous studies where subjects were passively moved by the robot, the HG employed during our experiment did not robustly guide subjects to eliminate the errors. The subjects had some freedom to select a timing strategy, while the compliant HG limited the overall timing error. We initially expected that this compliant HG would increase the opportunity of learning by imitation, as subjects would be active throughout the duration of the training. However, maybe the compliant HG gave subjects an erroneously good impression of their self-performance and they failed to consider the robot as the external force driving the good results; limiting the learning by imitation of the robotic action (Duarte and Reinkensmeyer 2015; Marchal-Crespo et al. 2015).

Considering the current results, EA and HG seems to activate different brain networks to foster motor learning. Following a neurological insult, such as a stroke, HG training is the most used technique by clinicians to promote recovery of their clients, by means of physical guidance of the affected limb (Marchal-Crespo and Reinkensmeyer 2009). This choice of therapy might not be suited for all clients since they usually present injuries to various brain areas. Thus, the current results further support the present trend in neurological rehabilitation where tailored rehabilitation to each individual’s potential for recovery and residual neural function is highly recommended to optimize chances of recovery (Hebert et al. 2016). Knowing which brain areas are affected by a neurological insult could guide clinicians in their choice of the best movement training rehabilitation for their clients in order to boost motor learning and recovery (Burke Quinlan et al. 2015).

### Limitations of the study

The present work recruited only young healthy subjects, limiting the generalization of our findings to older individuals. Indeed, two of our previous studies, using a similar timing task, found different behavioral changes in young (Milot et al. 2010) and elder participants (Bouchard et al. 2015) whereas young subjects’ timing performance improved with both HG and EA, but only HG training was beneficial in elderly people. Thus, it is possible that the patterns of brain activation during HG and EA training conditions would also differ between younger and older subjects. In addition, the fMRI results may have been affected by time-locking the stimulus with the TR (both were 2 s long), which can reduce the extent of hemodynamic response function high frequency sampling (Amaro and Barker 2006; Veltman et al. 2002), although that this concern is mitigated by the jitter in the response to the stimulus reflected in the timing error, the latter being of central importance to this study. Finally, the current study only evaluated brain activation associated with short-term learning with EA and HG training, hence it cannot be determined if the same or other brain networks would be activated with long-term learning induced by repeated practice sessions.

## Conclusion

This study evaluated whether different brain networks are activated when practicing a motor timing task with either haptic guidance or error amplification paradigms. The results revealed that during EA training, an error-detection system and fronto-parietal attentional/motor planning networks were strongly activated, likely because the task was challenging and elicited greater timing errors. During training with HG, error-processing brain structures were activated at the beginning, but with practice, a memory consolidation brain network was activated. Future work should explore the patterns of brain activation during longer or repeated practice sessions of EA and HG training for timing-based as well as spatial tasks. Having a better understanding of the neurophysiological underpinnings of motor learning processes during EA and HG training conditions might help implement patient-tailored robotic interventions in clinical research and practice.

## References

[CR1] Abdollahi F (2014). Error augmentation enhancing arm recovery in individuals with chronic stroke: a randomized crossover design. Neurorehabilit Neural Repair.

[CR2] Amaro E, Barker GJ (2006). Study design in fMRI: basic principles. Brain Cogn.

[CR3] Arsalidou M, Duerden EG, Taylor MJ (2013). The centre of the brain: topographical model of motor, cognitive, affective, and somatosensory functions of the basal ganglia. Hum Brain Mapp.

[CR4] Ashby FG, Crossley MJ (2012). Automaticity and multiple memory systems. Wiley Interdiscip Rev Cogn Sci.

[CR5] Battelli L, Pascual-Leone A, Cavanagh P (2007). The ‘when’ pathway of the right parietal lobe. Trends Cogn Sci.

[CR6] Bi S, Wan CX (2013). Comparison of the reaction time of wrist flexion and extension between patients with stroke and age-matched healthy subjects and correlation with clinical measures. Chin Med J (Engl).

[CR7] Bouchard AE, Corriveau H, Milot MH (2015). Comparison of haptic guidance and error amplification robotic trainings for the learning of a timing-based motor task by healthy seniors. Front Syst Neurosci.

[CR8] Bouchard AE, Corriveau H, Milot MH (2016). A single robotic session that guides or increases movement error in survivors post-chronic stroke: which intervention is best to boost the learning of a timing task?. Disabil Rehabil.

[CR9] Buccino G, Vogt S, Ritzl A, Fink GR, Zilles K, Freund HJ, Rizzolatti G (2004). Neural circuits underlying imitation learning of hand actions: an event-related fMRI study. Neuron.

[CR10] Burke Quinlan E (2015). Neural function, injury, and stroke subtype predict treatment gains after stroke. Ann Neurol.

[CR11] Carel C, Loubinoux I, Boulanouar K, Manelfe C, Rascol O, Celsis P, Chollet F (2000). Neural substrate for the effects of passive training on sensorimotor cortical representation: a study with functional magnetic resonance imaging in healthy subjects. J Cereb Blood Flow Metab.

[CR12] Caspers S, Zilles K, Laird AR, Eickhoff SB (2010). ALE meta-analysis of action observation and imitation in the human brain. NeuroImage.

[CR13] Chambon V, Sidarus N, Haggard P (2014). From action intentions to action effects: how does the sense of agency come about?. Front Hum Neurosci.

[CR14] Ciccarelli O (2005). Identifying brain regions for integrative sensorimotor processing with ankle movements. Exp Brain Res.

[CR15] Daly JJ, Fang Y, Perepezko EM, Siemionow V, Yue GH (2006). Prolonged cognitive planning time, elevated cognitive effort, and relationship to coordination and motor control following stroke. IEEE Trans Neural Syst Rehabil Eng.

[CR16] Daprati E, Sirigu A, Nico D (2010). Body and movement: consciousness in the parietal lobes. Neuropsychologia.

[CR17] Desmurget M, Reilly KT, Richard N, Szathmari A, Mottolese C, Sirigu A (2009). Movement intention after parietal cortex stimulation in humans. Science.

[CR18] DeWitt I, Rauschecker JP (2013). Wernicke’s area revisited: parallel streams and word processing. Brain Lang.

[CR19] Diedrichsen J, Hashambhoy Y, Rane T, Shadmehr R (2005). Neural correlates of reach errors. J Neurosci.

[CR20] Duarte JE, Reinkensmeyer DJ (2015). Effects of robotically modulating kinematic variability on motor skill learning and motivation. J Neurophysiol.

[CR21] Emken JL, Reinkensmeyer DJ (2005). Robot-enhanced motor learning: accelerating internal model formation during locomotion by transient dynamic amplification. IEEE Trans Neural Syst Rehabil Eng.

[CR22] Estevez N, Yu N, Brugger M, Villiger M, Hepp-Reymond MC, Riener R, Kollias S (2014). A reliability study on brain activation during active and passive arm movements supported by an MRI-compatible robot. Brain Topogr.

[CR23] Farrer C, Franck N, Georgieff N, Frith CD, Decety J, Jeannerod M (2003). Modulating the experience of agency: a positron emission tomography study. NeuroImage.

[CR24] Farrer C, Frey SH, Van Horn JD, Tunik E, Turk D, Inati S, Grafton ST (2008). The angular gyrus computes action awareness representations. Cereb Cortex.

[CR25] Freitas SM, Gera G, Scholz JP (2011). Timing variability of reach trajectories in left versus right hemisphere stroke. Brain Res.

[CR26] Friederici AD, Ruschemeyer SA, Hahne A, Fiebach CJ (2003). The role of left inferior frontal and superior temporal cortex in sentence comprehension: localizing syntactic and semantic processes. Cereb Cortex.

[CR27] Georgopoulos AP (2002). Cognitive motor control: spatial and temporal aspects. Curr Opin Neurobiol.

[CR28] Guadagnoli MA, Lee TD (2004). Challenge point: a framework for conceptualizing the effects of various practice conditions in motor learning. J Mot Behav.

[CR29] Halsband U, Lange RK (2006). Motor learning in man: a review of functional and clinical studies. J Physiol Paris.

[CR30] Hardwick RM, Rottschy C, Miall RC, Eickhoff SB (2013). A quantitative meta-analysis and review of motor learning in the human brain. NeuroImage.

[CR31] Hebert D (2016). Canadian stroke best practice recommendations: stroke rehabilitation practice guidelines, update 2015. Int J Stroke.

[CR32] Helie S, Ell SW, Ashby FG (2015). Learning robust cortico-cortical associations with the basal ganglia: an integrative review. Cortex.

[CR33] Heuer H, Luttgen J (2015). Robot assistance of motor learning: a neuro-cognitive perspective. Neurosci Biobehav Rev.

[CR34] Israely S, Carmeli E (2016). Error augmentation as a possible technique for improving upper extremity motor performance after a stroke—a systematic review. Top Stroke Rehabil.

[CR35] Izawa J, Shadmehr R (2011). Learning from sensory and reward prediction errors during motor adaptation. PLoS Comput Biol.

[CR36] Izawa J, Criscimagna-Hemminger SE, Shadmehr R (2012). Cerebellar contributions to reach adaptation and learning sensory consequences of action. J Neurosci.

[CR37] Jaeger L, Marchal-Crespo L, Wolf P, Riener R, Michels L, Kollias S (2014). Brain activation associated with active and passive lower limb stepping. Front Hum Neurosci.

[CR38] Japee S, Holiday K, Satyshur MD, Mukai I, Ungerleider LG (2015). A role of right middle frontal gyrus in reorienting of attention: a case study. Front Syst Neurosci.

[CR39] Kana RK, Ammons CJ, Doss CF, Waite ME, Kana B, Herringshaw AJ, Ver Hoef L (2015). Language and motor cortex response to comprehending accidental and intentional action sentences. Neuropsychologia.

[CR40] Kessler K, Biermann-Ruben K, Jonas M, Siebner HR, Baumer T, Munchau A, Schnitzler A (2006). Investigating the human mirror neuron system by means of cortical synchronization during the imitation of biological movements. NeuroImage.

[CR41] Kim HF, Hikosaka O (2015). Parallel basal ganglia circuits for voluntary and automatic behaviour to reach rewards. Brain.

[CR42] Kim YT (2011). Neural correlates related to action observation in expert archers. Behav Brain Res.

[CR43] Land MF (2014). Do we have an internal model of the outside world?. Philos Trans R Soc Lond B Biol Sci.

[CR44] Loubinoux I (2001). Within-session and between-session reproducibility of cerebral sensorimotor activation: a test–retest effect evidenced with functional magnetic resonance imaging. J Cereb Blood Flow Metab.

[CR45] Luck D, Danion JM, Marrer C, Pham BT, Gounot D, Foucher J (2010). The right parahippocampal gyrus contributes to the formation and maintenance of bound information in working memory. Brain Cogn.

[CR46] Makino H, Hwang E, Hedrick N, Komiyama T (2016). Circuit mechanisms of sensorimotor learning. Neuron.

[CR47] Marchal-Crespo L, Reinkensmeyer DJ (2009). Review of control strategies for robotic movement training after neurologic injury. J Neuroeng Rehabil.

[CR48] Marchal-Crespo L, van Raai M, Rauter G, Wolf P, Riener R (2013). The effect of haptic guidance and visual feedback on learning a complex tennis task. Exp Brain Res.

[CR49] Marchal-Crespo L, Schneider J, Jaeger L, Riener R (2014). Learning a locomotor task: with or without errors?. J Neuroeng Rehabil.

[CR50] Marchal-Crespo L, Bannwart M, Riener R, Vallery H (2015). The effect of haptic guidance on learning a hybrid rhythmic-discrete motor task. IEEE Trans Haptics.

[CR51] Marchal-Crespo L, Michels L, Jaeger L, Lopez-Oloriz J, Riener R (2017). Effect of error augmentation on brain activation and motor learning of a complex locomotor. Task Front Neurosci.

[CR52] Marchal-Crespo L, Rappo N, Riener R (2017). The effectiveness of robotic training depends on motor task characteristics. Exp Brain Res.

[CR53] Milot MH, Marchal-Crespo L, Green CS, Cramer SC, Reinkensmeyer DJ (2010). Comparison of error-amplification and haptic-guidance training techniques for learning of a timing-based motor task by healthy individuals. Exp Brain Res.

[CR54] Miscio G, Pisano F, Del Conte C, Colombo R, Schieppati M (2006). Concurrent changes in shortening reaction latency and reaction time of forearm muscles in post-stroke patients. Neurol Sci.

[CR55] Morris LS (2016). Fronto-striatal organization: defining functional and microstructural substrates of behavioural flexibility. Cortex.

[CR56] Nadig KG, Jancke L, Luchinger R, Lutz K (2010). Motor and non-motor error and the influence of error magnitude on brain activity. Exp Brain Res.

[CR57] Nakamura K, Santos GS, Matsuzaki R, Nakahara H (2012). Differential reward coding in the subdivisions of the primate caudate during an oculomotor task. J Neurosci.

[CR58] O’Connell RG (2007). The role of cingulate cortex in the detection of errors with and without awareness: a high-density electrical mapping study. Eur J Neurosci.

[CR59] Owen AM, Milner B, Petrides M, Evans AC (1996). A specific role for the right parahippocampal gyrus in the retrieval of object-location: a positron emission tomography study. J Cogn Neurosci.

[CR60] Park JW, Kim YH, Jang SH, Chang WH, Park CH, Kim ST (2010). Dynamic changes in the cortico-subcortical network during early motor learning. NeuroRehabilitation.

[CR61] Patton JL, Mussa-Ivaldi FA (2004). Robot-assisted adaptive training: custom force fields for teaching movement patterns. IEEE Trans Bio-med Eng.

[CR62] Patton JL, Stoykov ME, Kovic M, Mussa-Ivaldi FA (2006). Evaluation of robotic training forces that either enhance or reduce error in chronic hemiparetic stroke survivors. Exp Brain Res.

[CR63] Price CJ (2010). The anatomy of language: a review of 100 fMRI studies published in 2009. Ann N Y Acad Sci.

[CR64] Radovanovic S (2002). Comparison of brain activity during different types of proprioceptive inputs: a positron emission tomography study. Exp Brain Res.

[CR65] Roux FE, Miskin K, Durand JB, Sacko O, Rehault E, Tanova R, Demonet JF (2015). Electrostimulation mapping of comprehension of auditory and visual words. Cortex.

[CR66] Rugg MD, King DR (2017). Ventral lateral parietal cortex and episodic memory retrieval. Cortex.

[CR67] Rushworth MF, Krams M, Passingham RE (2001). The attentional role of the left parietal cortex: the distinct lateralization and localization of motor attention in the human brain. J Cogn Neurosci.

[CR68] Schultz W (2016). Reward functions of the basal ganglia. J Neural Transm (Vienna).

[CR69] Scolari M, Seidl-Rathkopf KN, Kastner S (2015). Functions of the human frontoparietal attention network: evidence from neuroimaging. Curr Opin Behav Sci.

[CR70] Shadmehr R, Holcomb HH (1997). Neural correlates of motor memory consolidation. Science.

[CR71] Sirigu A, Daprati E, Pradat-Diehl P, Franck N, Jeannerod M (1999). Perception of self-generated movement following left parietal lesion. Brain.

[CR72] Taylor SF, Stern ER, Gehring WJ (2007). Neural systems for error monitoring: recent findings and theoretical perspectives. Neuroscientist.

[CR73] Tseng YW, Diedrichsen J, Krakauer JW, Shadmehr R, Bastian AJ (2007). Sensory prediction errors drive cerebellum-dependent adaptation of reaching. J Neurophysiol.

[CR74] van Asseldonk EH, Wessels M, Stienen AH, van der Helm FC, van der Kooij H (2009). Influence of haptic guidance in learning a novel visuomotor task. J Physiol Paris.

[CR75] Veltman DJ, Mechelli A, Friston KJ, Price CJ (2002). The importance of distributed sampling in blocked functional magnetic resonance imaging designs. NeuroImage.

[CR76] Weiller C (1996). Brain representation of active and passive movements. NeuroImage.

